# Identification of Side Chain Oxidized Sterols as Novel Liver X Receptor Agonists with Therapeutic Potential in the Treatment of Cardiovascular and Neurodegenerative Diseases

**DOI:** 10.3390/ijms24021290

**Published:** 2023-01-09

**Authors:** Na Zhan, Boyang Wang, Nikita Martens, Yankai Liu, Shangge Zhao, Gardi Voortman, Jeroen van Rooij, Frank Leijten, Tim Vanmierlo, Folkert Kuipers, Johan W. Jonker, Vincent W. Bloks, Dieter Lütjohann, Marcella Palumbo, Francesca Zimetti, Maria Pia Adorni, Hongbing Liu, Monique T. Mulder

**Affiliations:** 1 Key Laboratory of Marine Drugs, Ministry of Education, School of Medicine and Pharmacy, Ocean University of China, Qingdao 266003, China; 2 Department of Internal Medicine, Erasmus Medical Center, 3015 CN Rotterdam, The Netherlands; 3 Department of Neuroscience, Biomedical Research Institute, Hasselt University, 3500 Hasselt, Belgium; 4 School for Mental Health and Neuroscience, Maastricht University, 6229 ER Maastricht, The Netherlands; 5 Department of Pediatrics, University Medical Center Groningen, University of Groningen, 9713 GZ Groningen, The Netherlands; 6 European Research Institute for the Biology of Ageing (ERIBA), University Medical Center Groningen, University of Groningen, 9713 GZ Groningen, The Netherlands; 7 Institute of Clinical Chemistry and Clinical Pharmacology, University Hospital Bonn, 53105 Bonn, Germany; 8 Department of Food and Drug, University of Parma, 43124 Parma, Italy; 9 Unit of Neurosciences, Department of Medicine and Surgery, University of Parma, 43125 Parma, Italy

**Keywords:** oxidized sterols, LXR agonists, cardiovascular disease, Alzheimer’s disease, cholesterol efflux

## Abstract

The nuclear receptors—liver X receptors (LXR α and β) are potential therapeutic targets in cardiovascular and neurodegenerative diseases because of their key role in the regulation of lipid homeostasis and inflammatory processes. Specific oxy(phyto)sterols differentially modulate the transcriptional activity of LXRs providing opportunities to develop compounds with improved therapeutic characteristics. We isolated oxyphytosterols from *Sargassum fusiforme* and synthesized sidechain oxidized sterol derivatives. Five 24-oxidized sterols demonstrated a high potency for LXRα/β activation in luciferase reporter assays and induction of LXR-target genes *APOE*, *ABCA1* and *ABCG1* involved in cellular cholesterol turnover in cultured cells: methyl 3β-hydroxychol-5-en-24-oate (**S1**), methyl (3β)-3-aldehydeoxychol-5-en-24-oate (**S2**), 24-ketocholesterol (**S6**), (3β,22E)-3-hydroxycholesta-5,22-dien-24-one (**N10**) and fucosterol-24,28 epoxide (**N12**). These compounds induced *SREBF1* but not SREBP1c-mediated lipogenic genes such as *SCD1*, *ACACA* and *FASN* in HepG2 cells or astrocytoma cells. Moreover, **S2** and **S6** enhanced cholesterol efflux from HepG2 cells. All five oxysterols induced production of the endogenous LXR agonists 24(S)-hydroxycholesterol by upregulating the *CYP46A1*, encoding the enzyme converting cholesterol into 24(S)-hydroxycholesterol; **S1** and **S6** may also act via the upregulation of desmosterol production. Thus, we identified five novel LXR-activating 24-oxidized sterols with a potential for therapeutic applications in neurodegenerative and cardiovascular diseases.

## 1. Introduction

Liver X receptors (LXRs) α (*NR1H3*) and β (*NR1H2*) are cholesterol-sensing nuclear receptors and play key roles in transcriptional control of lipid metabolism and in modulating immune and inflammatory responses [1,2] by controlling the expression of specific target genes [3,4]. Based on these functions LXRs have been extensively explored therapeutic targets in the treatment of neurodegenerative [5,6,7] and cardiometabolic diseases [8,9], conditions involving dysregulated cholesterol homeostasis and inflammation. LXRs form obligatory heterodimers with retinoid X receptor (RXR) α. LXRs do not sense cholesterol itself but can be activated by various cholesterol metabolites such as natural oxysterols [10], including 22(*R*)-hydroxycholesterol (22R-OHC), 27-hydroxycholesterol (27-OHC), 24(*S*)-hydroxycholesterol (24S-OHC), and cholestenoic acid [11] as well as by intermediates of the cholesterol biosynthesis pathway such as 24(*S*),25-epoxycholesterol, zymosterol and desmosterol, the latter being the most abundant endogenous LXR activator [9,12,13,14,15]. In the non-liganded state, LXR acts as a repressor, whereas in the liganded state it acts as an activator of target gene expression, thus serving as a molecular switch for those genes that contain an LXR-responsive element (LXRE) [16,17,18,19,20].

The central nervous system (CNS) requires a complex and delicately balanced cholesterol metabolism to maintain homeostasis and, thereby, adequate neuronal functions. An imbalance in cholesterol homeostasis has been implicated in a number of neurodegenerative diseases including Alzheimer’s disease (AD), Parkinson’s disease (PD), Huntington’s disease (HD), and multiple sclerosis (MS). Cholesterol is unable to pass the blood-brain barrier (BBB), and therefore, brain cholesterol needs to be synthesized locally [21]. Oxysterols and the enzymes that catalyze their synthesis, such as cholesterol 24-hydroxylase (CYP46A1) [22,23], have been found to be altered in the brain of AD patients [24,25], indicating the involvement of LXRs. Due to their function as cholesterol sensors and their involvement in the regulation of lipid metabolism and inflammation, LXRs are critical in the maintenance of CNS homeostasis. We and others reported that cognition in AD mice can be improved by enhancing cholesterol turnover in the brain via LXR-activation using the synthetic agonist T0901317 [26,27]. LXRs have also been implicated in the metabolic and inflammatory pathways that are involved in the pathogenesis of cardiovascular and metabolic diseases [6,7,28]. For instance, LXR interferes with atherogenesis by affecting signaling pathways in macrophages, either by promoting reverse cholesterol transport (RCT), thereby limiting cholesterol deposition, or through the inhibition of pro-inflammatory gene expression, thereby reducing lesion-associated inflammation [28,29]. In addition, natural LXR agonists that activate target genes related to cholesterol turnover have been found to inhibit the activation of *SREBF1* the gene encoding the sterol regulatory element-binding transcription factor (SREBP1c) and, consequently, lipogenesis [30,31].

However, the therapeutic application of synthetic pan-LXR agonists is hindered by unwanted side effects, particularly hypertriglyceridemia and hepatic steatosis. These side effects are thought to result from hepatic LXRα activation, leading to increased transcription of SREBP1c in the liver [32]. The high similarity in the ligand binding domain (LBD) of LXRα and LXRβ has likely hindered the development of LXRβ-specific ligands. Notably, LXRα and LXRβ have distinct tissue distributions. LXRα is highly expressed in metabolically active tissues and cell types, including the liver, intestine, adipose tissue and macrophages [33], whereas LXRβ mainly expressed in the nervous system and endocrine system [34,35]. Evidently, tissue-specific LXR activation may circumvent this problem. For example, selective targeting of macrophages was reported to reduce atherosclerosis without inducing hepatic lipogenesis [36]. Oxyphytosterols share a close structural similarity with the endogenous LXRα/β ligands, the oxysterols [37]. We previously reported that (oxy)phytosterols, unlike cholesterol, are able to cross the BBB [38,39,40]. These naturally occurring compounds have been shown to modulate the activity of LXRs and could offer potential therapeutic efficacy, while suppressing SREBP1c target genes to minimize side effects [13], such as hypertriglyceridemia [41]. We found that the autoxidation product of fucosterol [42], 24(*S*)-saringosterol, present in the brown seaweed *Sargassum fusiforme* (*S. fusiforme*), preferentially activates LXRβ [43]. 24(*S*)-saringosterol differs from the LXR endogenous ligand 24(*S*)-OHC by the presence of an additional ethylene group on C-24. Recently, we reported that dietary supplementation with the 24(*S*)-saringosterol-containing lipid extract of *S. fusiforme* or pure 24(*S*)-saringosterol prevents memory decline in an AD animal model, without inducing the side effects induced by synthetic pan-LXR agonists [44,45]. Consistently, saringosterol treatment reduced atherosclerotic plaque burden and reduced plasma cholesterol concentrations without having undesirable adverse hepatic effects in APOE-knockout mice fed an atherogenic diet [46]. These studies highlighted the potential of oxyphytosterols in the treatment of AD.

In the current study, we obtained and tested a series of new sidechain mono-oxidated sterols as potential therapeutic candidates for the modulation of cholesterol homeostasis via LXR activation. We first investigated the LXRα/β activating capacity of oxidized sterol derivatives, which were either isolated from *S*. *fusiforme* or semi-synthesized from hyodeoxycholic acid. Furthermore, we determined their modulating effect on the expression of LXR-response genes in different cell types in vitro. We examined the effect of candidates on cholesterol metabolism by assessing the effect on endogenous cholesterol synthesis and efflux. Together, this resulted in five 24-oxidized sterols with high potency to stimulate LXRα and LXRβ transcriptional activity without inducing lipogenic genes.

## 2. Results

### 2.1. Chemistry

**Figure 1:**
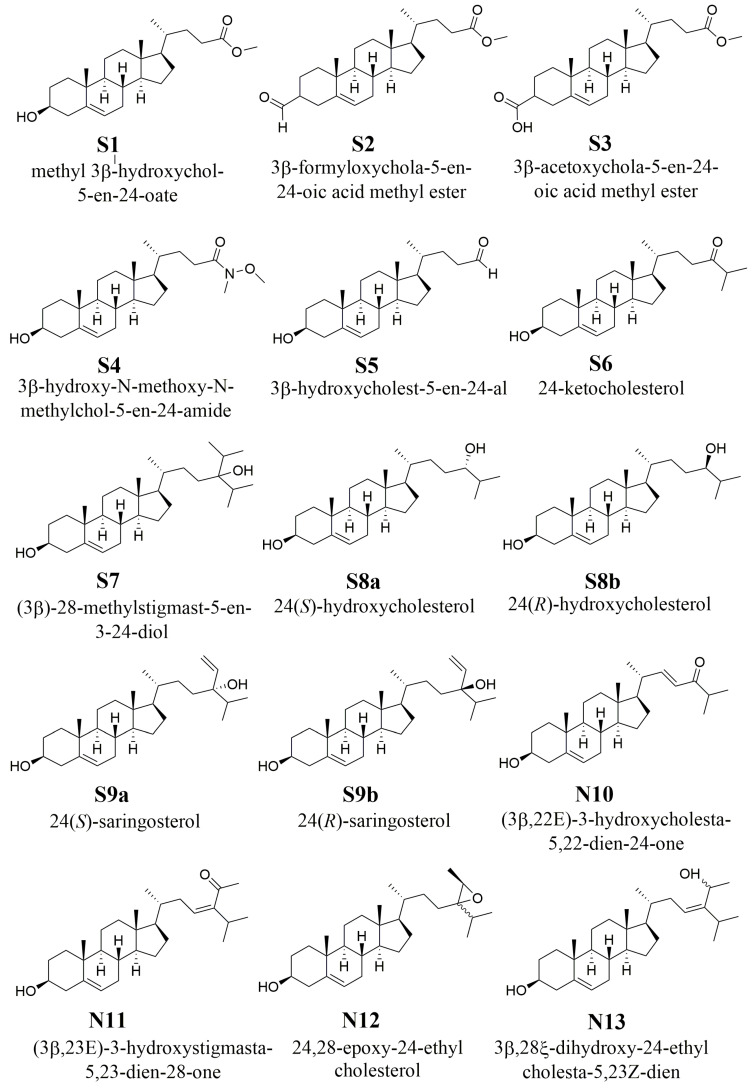
Chemical structures of **S1-S9** and **N10-N1**.

Natural and semi-synthetic phytosterol derivatives characterized by the presence of side-chain oxidation have been shown to be able to modulate LXR activity. In the frame of a vast research project aimed at the synthesis of phytosterol derivatives bearing oxidized sidechains, we designed a synthetic route to generate oxysterols and test their LXR-activating properties. Nine oxysterol derivatives were efficiently synthesized (**S1-S9**), all endowed with an oxidized group at their side chain. With the aim to continue the search for sidechain oxidized plant oxysterols we included four naturally occurring oxidized sterol derivatives (**N10-N13**), other than saringosterol, isolated from *Sargassum fusiforme* (Figure 1).

### 2.2. LXRα and LXRβ Activation

**Figure 2:**
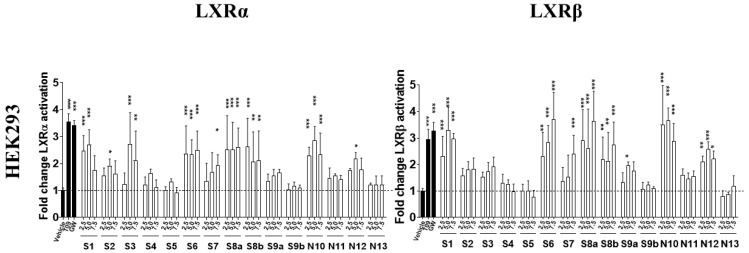
Effects of sidechain oxidized sterols on transcriptional activity of LXRα and β in HEK 293 cells. Using the luciferase reporter assay the capacity of oxidized sterols on LXR-mediated transcription was determined. Transfected cells were incubated with different concentrations of oxysterols (2.5, 5.0 and 7.5 µM) for 24 h. T0901317 (T09, 1 µM) and GW3965 (GW, 5 µM) were used as positive controls. Data represent the mean ± SD of three separate experiments, each performed in triplicate (n = 9). Significance is compared to the control (DMEM/F-12 medium with EtOH or DMSO) value: * *p* ≤ 0.05, ** *p* ≤ 0.01, *** *p* ≤ 0.001.

The natural and synthesized side chain oxidized sterols were initially assessed for their LXRα and LXRβ transactivating capacity in vitro using a dual luciferase reporter assay in HEK293 cells (Figure 2). Among the 13 compounds, derivatives **S1**, **S6**, **S7**, **N10**, and **N12** displayed the highest efficacy for LXRα and LXRβ activation, comparable to 24(S/R)-OHC (**S8a/b**) and 24(*S*)-saringosterol (**S9a**) (Table 1). **S2**, **S3** and **N11** displayed limited effects, and **S4**, **S5**, **S9b**, and **N13** activated LXRs hardly or not at all.

In CCF-STTG1 astroglial cells, besides 24(*S*/*R*)-OHC and 24(*S*)-saringosterol, only **S6** significantly activated LXRα and LXRβ. **S1** significantly activated LXRα, but not LXRβ (Figure S1). In CHME3 microglial cells, LXRα and LXRβ were activated most notably by **S1**, **S6**, and **N10**, also by **S2**, **S3** and **S4**, and hardly by **S5, S7, N11** and **N13**. In SH-SY5Y neuroblastoma cells, the effect of the compounds on LXR activation was more pronounced than in glial cells and was mostly dose-dependent. **S1**, **S2**, **S6**, **N10** as well as **N12**, in addition to **S8a/b** and **S9a**, exhibited a moderate to strong capacity for the activation of both LXRα and LXRβ, while no effect was observed for **S4**, **S5**, **S7**, **S9b**, and **N13** (Figure S1)

In HepG2 cells, the most potent agonists for LXRα and LXRβ were **S1**, **S2**, **S6**, and **N10**, while **N12** activated LXRβ predominantly. As compared to the CNS cell lines, relatively high concentrations were required to activate LXRs in these liver-derived cells (Figure S1).

Although LXRs form permissive heterodimers with RXR that can be activated by ligands for LXR or RXR [47,48], weak effects of the compounds on RXR were detected in CHME3 and CCF-STTG1, while the direct activity on RXR in SH-SY5Y was absent (Figure S2). On the other hand, in HepG2 cells, relatively high concentrations of **S1**, **S2**, **S6**, **N10** and **N12** did activate RXR.

Using this approach, we identified five new candidates **S1**, **S2**, **S6**, **N10**, and **N12** showing equal or higher activation efficiency of LXRs than **S8a** and **S9a**. **S4**, **S5**, **S7**, and **N13** displayed no effects.

### 2.3. LXR-Target Gene Expression in CNS Cell Lines

**Figure 3:**
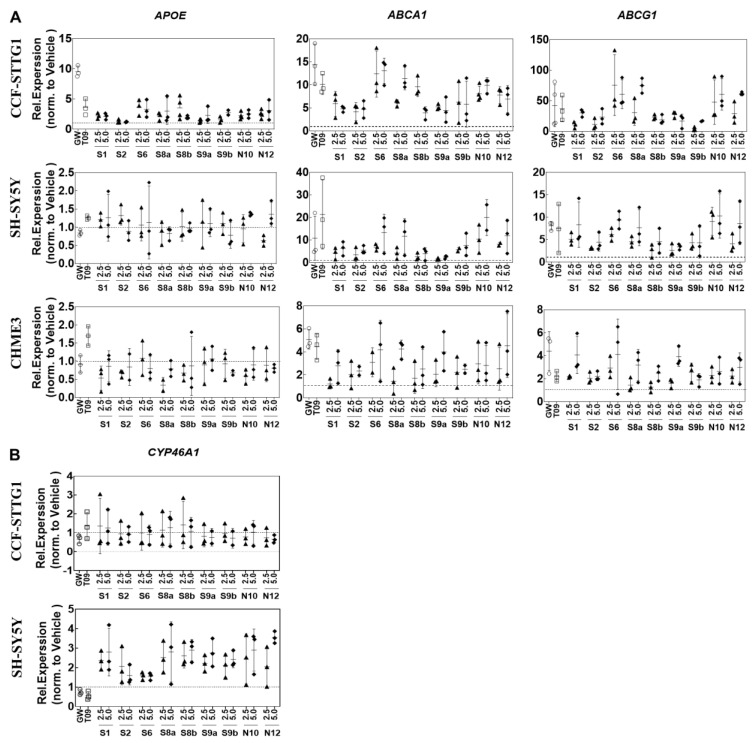
Differential effects of five oxidized sterols on cholesterol turnover genes and *CYP46A1* mRNA levels. (**A**) The expression of *APOE, ABCA1* and *ABCG1* in CCF-STTG1, CHME3 and SH-SY-5Y cells. (**B**) *CYP46A1* expression in CCF-STTG1 and SH-SY-5Y cells. Cells were incubated with 2.5 or 5.0 μM of oxidized sterols and 1 μM T0901317 (T09) and 5 μM GW3965 (GW) compound for 24 h. Total RNA was isolated from the cells and analyzed by qPCR as described in Materials and Methods. Gene expression was normalized to mean of the most stable housekeeping genes (*SDHA, B2M, ACTB* and *HPRT1*) and expressed as Relative Expression compared to the vehicle control. The values are presented as the means of three experiments ± SD (*n* = 3).

Next, we assessed the effects of the most potent LXR-activating oxidized sterols on the expression of selected LXR-target genes involved in cholesterol homeostasis, starting with efflux pathway-related genes, i.e., *APOE*, *ABCA1,* and *ABCG1* [49,50] (Figure 3). Incubation of CCF-STTG1 cells with **S1**, **S2**, **S6**, **N10**, **N12**, and **S8a/b** and **S9a/b** resulted in increased *APOE* mRNA levels, while no effect was observed in CHME3 and SH-SY5Y cells (Figure 3A). In line with these results, T0901317 (1μM) and GW3965 (5.0 μM) induced *APOE* expression in CCF-STTG1, but not in the other two cell types.

In CCF-STTG1 cells **S6**, **N10**, and **N12** upregulated the expression of *ABCA1*, and *ABCG1,* more than or comparable to **S8a** and **S9a**. Comparable results were obtained in SH-SY5Y cells (Figure 3A). The expression of *ABCG1* was induced to a higher extent in CCF-STTG1 than in SH-SY5Y or CHME3 cells.

Cholesterol can be converted into 24S-OHC via hydroxylation of cholesterol by CYP46A1 in neurons. In line with the predominant neuronal expression of *CYP46A1* [51], its expression was found to be the highest (approximately three-fold) in SH-SY5Y cells as compared to glial cells (Figure 3B). **S1**, **S2**, **S6**, **N10**, **N12**, and **S8a/b** and **S9a/b** increased *CYP46A1* expression in SH-SY5Y cells. In CCF-STTG1 cells, on the other hand, its expression was hardly affected.

### 2.4. Cell-Specific Discrimination of Oxysterols Selectively Regulate SREBF1 and Its Downstream Lipogenic Genes

**Figure 4:**
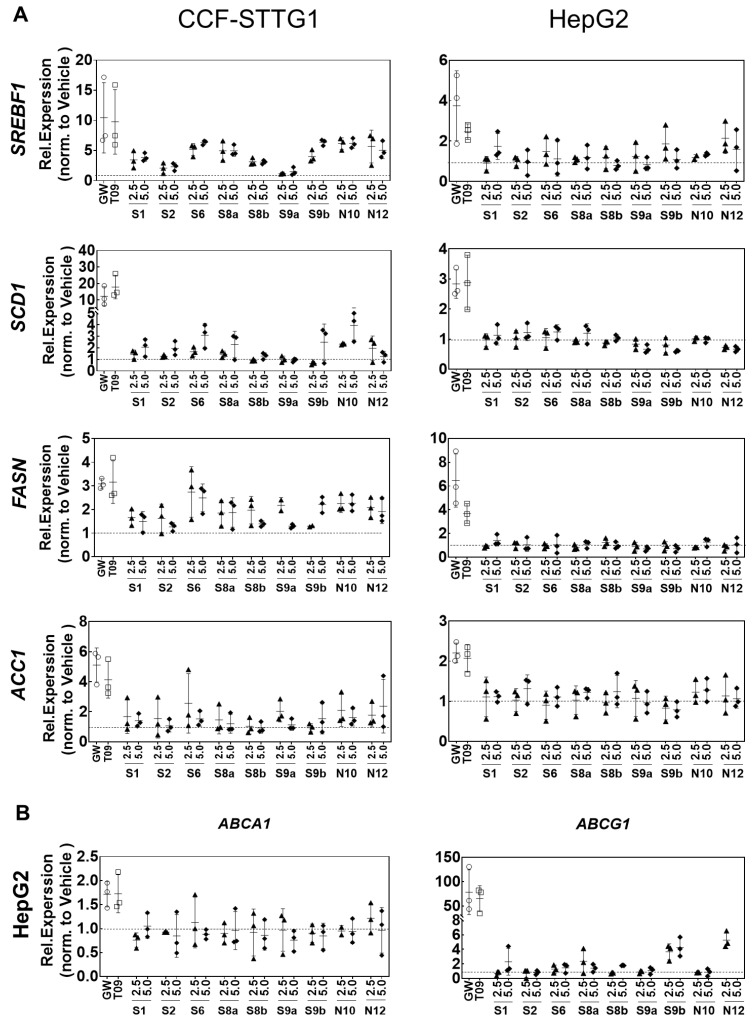
Effects of LXR-activating oxidized sterols on the gene’s expression of lipogenesis. (**A**) *SREBF1, SCD1, FASN* and *ACACA* expression in CCG-STTG1 or HepG2 cells. (**B**) *ABCA1* and *ABCG1* expression in *HepG2 cells.* Cells were incubated with EtOH (0.1%, *v/v*), DMSO (0.1%, *v/v*), 1 μM T0901317 (T09), 5 μM GW3965 (GW), 2.5 or 5.0 μM of oxysterols for 24 h and gene expression was assessed by qPCR. Gene expression was normalized to the most stable housekeeping genes (SDHA, B2M, ACTB, and HPRT1) and expressed as Relative. Expression compared to the vehicle control (EtOH or DMSO) given as means of three experiments ± SD (*n* = 3).

Most synthetic LXR agonists that have been generated, have limited clinical application because of the consequences of LXRα-induced hepatic upregulation of the expression of lipogenic genes including *SREBF1* [32,52] and its target genes involved in lipogenesis, i.e., stearoyl-CoA desaturase-1 (*SCD1*), fatty acid synthase (*FASN*) and Acetyl-CoA carboxylase (*ACACA*) [53,54]. Notably, the treatment of CCF-STTG1 with increasing concentrations of oxysterols led to coordinate increases in LXR-dependent SREBP1c pathways. As a consequence, genes involved in cholesterol efflux and lipid synthesis genes were induced (Figure 3 and Figure 4). However, oxysterols suppress genes involved in cholesterol and fatty acid synthesis in HepG2 cells. The data presented in Figure 4 show that in HepG2 cells, none of the oxidized sterols upregulated the expression of *SREBF1*, *SCD1, FASN or ACACA*. **N12** and 24(*R*)-saringosterol showed a limited effect on the expression of *SREBF1*. Compared to the other cell types, HepG2 was less responsive regarding the induction of *ABCA1* and *ABCG1* by oxysterols. In particular, **N12** and **S9b** induced the expression of *ABCG1* in HepG2 cells, however, to a less extent than T0901317 and GW3965 (Figure 4B).

### 2.5. Effect on Cholesterol Efflux

**Figure 5:**
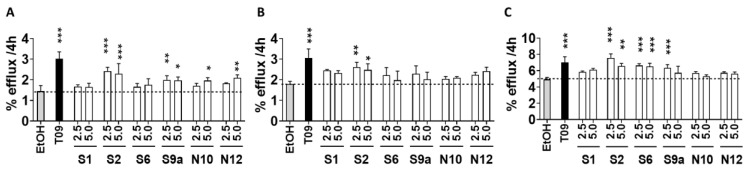
Effect of LXR ligands on cholesterol efflux from HepG2 cells. HepG2 cells were loaded for 24 h with 2 μCi/mL [^3^H] cholesterol in the presence of 1% FCS and then incubated for 20 h with vehicle (ethanol), 2.5 μM or 5 μM LXR ligands. Cholesterol efflux was promoted to (**A**) human APOA1 (10 µg/mL), (**B**) human HDL (12.5 µg/mL), and (**C**) a pool of serum from normolipidemic individuals at 2% (*v/v*) for 4 h and assayed as described under Methods. Each cell treatment was performed in triplicate and data are expressed in percentage as mean ± SD (*n* = 3). Significance is compared to the control (EtOH) value: * *p* ≤ 0.05, ** *p* ≤ 0.01, *** *p* ≤ 0.001.

All selected oxidized sterols significantly affected cholesterol efflux to APOA1, except for **S1** and **S6** (Figure 5A). The APOA1 mediated efflux induced by **N10** and **N12** was significant at the highest concentration (5 µM) exclusively. Only **S2** increased cholesterol efflux to HDL (Figure 5B). We also observed a significant increase in cholesterol efflux to human serum after incubation with **S2** and **S6** (Figure 5C). **S9a** also significantly stimulated serum-mediated efflux at 2.5 µM. Overall, **S2** showed the highest capacity to induce cholesterol efflux. T0901317 at a concentration of 1 μM significantly increased cholesterol efflux from HepG2 cells in the presence of APOA1, HDL, or human serum as a cholesterol acceptor (Figure 5).

**Figure 6:**
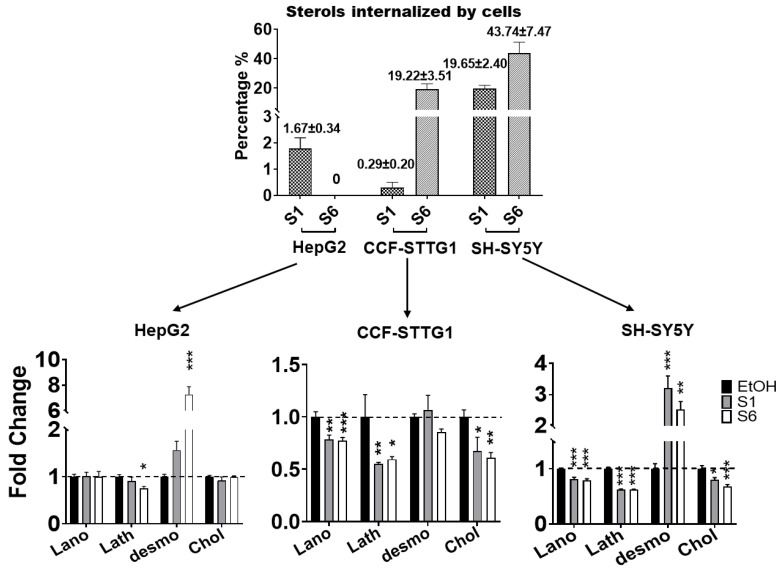
**S1** and **S6** regulate cholesterol biosynthesis in a cell type-specific manner. Lanosterol (Lano), lathosterol (Lath), desmosterol (Desmo), and cholesterol (Chol) were quantified in S1 or S6 loaded cells (HepG2, CCF-STTG1 and SHSH-5Y) using GC-MS method. Fold change compared to the vehicle control. Each bar represents the mean ± SD of three separate experiments, each performed in triplicate (*n* = 9). Significance is compared to the control (EtOH) value: * *p* ≤ 0.05, ** *p* ≤ 0.01, *** *p* ≤ 0.001.

We then questioned if the semisynthetic oxysterols **S1** and **S6** regulate cholesterol homeostasis via negative feedback regulation of 3-hydroxy-3-methylglutaryl-CoAreductase (HMGCR), the rate-limiting enzyme of cholesterol biosynthesis, similar to endogenous oxysterols such as 27-OHC or 24-OHC [55,56]. Therefore, we first assessed the internalization of **S1** and **S6** by different cell types. The internalization of **S1** by HepG2 (1.67%) and CCF-STTG1 (0.29%) cells was rather low. Yet, the internalization by SH-SY5Y cells was more efficient (19.22%). The uptake of **S6** was high in both CCF-STTG1 (19.65%) and SH-SY5Y (43.74%) cells; no **S6** could be detected in HepG2 cells while the amount remaining in the medium was reduced. To determine the effect of **S1** and **S6** on cholesterol synthesis, we measured their effect on cellular levels of cholesterol and its precursors, lanosterol, lathosterol, and desmosterol. Although **S6** significantly increased desmosterol concentrations, it did not affect the cholesterol concentration in HepG2 cells. We found that both **S1** and **S6** significantly increased desmosterol concentrations in SH-SY5Y cells, but not in CCF-STTG1 cells (Figure 6). **S6** reduced intracellular levels of cholesterol in CCF-STTG1 and SH-SY5Y, as well as levels of lathosterol and, to a lesser extent, lanosterol.

### 2.6. LXR-Activating Oxidized Sterols Down-Regulate DHCR7 and DHCR24 Gene Expression

**Figure 7:**
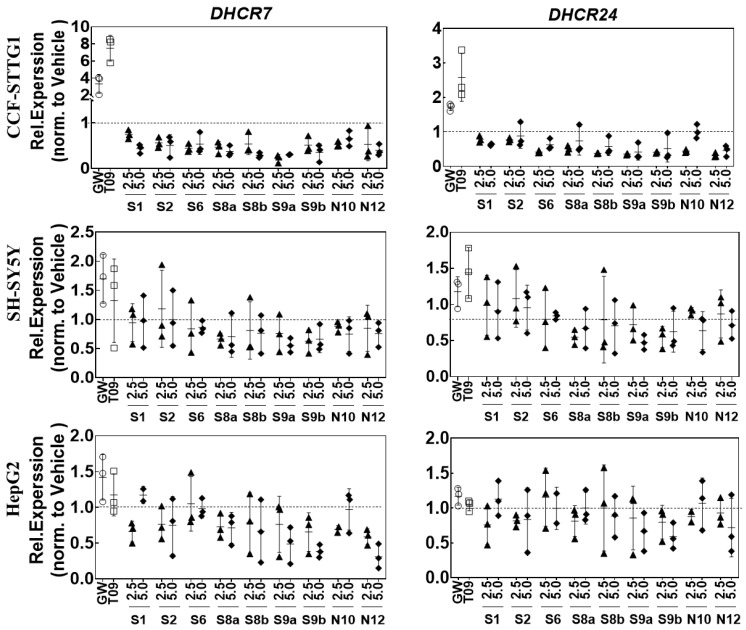
Effects of oxysterols on *DHCR7* and *DHCR24* mRNA levels. Cells were incubated with 2.5 or 5.0 μM of 24-oxidized sterols and 1 μM T0901317 (T09) and 5 μM GW3965 (GW) for 24 h. Gene expression was determined by qPCR and normalized to the most stable housekeeping genes (*SDHA*, *B2M*, *ACTB* and *HPRT1*) and expressed as Relative Expression compared to the vehicle control. The values are presented as the means of three experiments ± SD (*n* = 3).

Accumulation of desmosterol may result from alterations in the activity of 24-dehydrocholesterol reductase (DHCR24) or 7-dehydrocholesterol reductase (DHCR7). Therefore, we next examined the expression of the genes encoding these enzymes. Expression of *DHCR7* was decreased in CCF-STTG1 cells by all compounds, and the expression of *DHCR24* was decreased by **S1**, **N12**, **S8a/b** and **S9a/b**. Conversely, the treatment of cells with the synthetic LXR ligands T0901317 and GW3965 increased the expression of these two genes. 24(*S*)/24(*R*)-OHC and 24(*S*)/24(*R*)-saringosterol showed minor effects on *DHCR7* and *DHCR24* in SH-SY5Y cells. **S2**, **N12**, **S8a/b** and **S9a/b** decreased the expression of *DHCR7*, while **S9a/b** increased the expression of *DHCR24* in HepG2 cells (Figure 7).

## 3. Discussion

Upon preliminary examination of thirteen oxidized sterols for their LXRα- and LXRβ-activating capacity we identified three novel semi-synthesized and two natural candidates: **S1**, **S2**, **S6**, **N10** and **N12**. The naturally oxidized sterols **N10** and **N12** that were isolated from *Sargassum fusiforme* showed the highest LXR-activating capacity while the semi-synthesized **S1**, **S2** and **S6**, displayed an LXR activating capacity comparable to or even higher than the endogenous agonist **S8a** and the exogenous agonist **S9a**. These five oxidized sterols that activated LXRα and LXRβ were found to regulate the expression of a number of LXR-target genes involved in cholesterol homeostasis in a cell-type specific manner. In line with the literature, the LXR-activating oxidized oxysterols did not induce the SREBP1c target genes *FASN*, *SCD1* and *ACACA* in HepG2 cells [30,31]. In contrast, these five oxysterols did induce the lipogenic pathway in CCF-STTG1 cells. The processing of SREBP is suppressed by indirect oxysterol-mediated enhancement of SREBP cleavage-activating protein (SCAP) binding to insulin-induced gene (INSIG) proteins (1 or 2), thereby preventing the movement of the SCAP-SREBP complex to the Golgi apparatus where SREBPs are subsequently activated [30]. **S6**, which induced a significant increase in endogenous LXR agonist desmosterol, also did not upregulate SREBP1c expression in hepatic cells. Accordingly, desmosterol was found to suppress SREBP activation by directly binding to SCAP [13]. Thus, while oxysterols and desmosterol activate LXRs, they suppress SREBP1c-induced hypertriglyceridemia [13]. The five oxysterols and desmosterol being LXR agonists may balance lipid homeostasis via reciprocal actions on LXRs and SREBP activities.

The relatively weak LXR activating capacity of **N11** and **N13** might be explained by the fact that these compounds have an oxidation group at C28 rather than at C24. It has been demonstrated that the mono-oxidation of specific sites on the cholesterol side chain is necessary for high-affinity binding to both LXRs [57]. We found that a large, saturated group such as in **S7**, or a plane structure formed by amide such as in **S4** could make the side chain less flexible and thereby decrease rotational freedom. Such large, saturated groups appear to be sufficient to partially attenuate the ability to interact with LXR binding. **S1**, **S2** and **S3** differ in molecular structure by a -OH, -COOH, or -OCOCH3 at the C-3 position and the capacity to activate LXRs decreased with the increasing size of the substituents. The structure may thus affect the binding efficiency of the molecule to the ligand-binding domain.

Another reason for the differences in the activation of LXRs may be due to variable intracellular concentrations of these oxysterols. Their different structures may affect the efficiency of transport across cell membranes, possibly resulting in low effective intracellular concentrations to bind LXRs. In addition, the way oxysterols are taken up determines their activity. Receptor-mediated endocytosis results in a different subcellular presence as compared to passive diffusion. The differential uptake of the oxidized sterols may also occur through the binding of lipoproteins that are present in the culture medium and consequently depend on the presence of specific cellular receptors. We found **S1** to be unable to activate LXRβ in CCF-STTG1 cells, which was likely due to its extremely low internalization (0.29% ± 0.2) in this cell line. Both **S1** and **S6**, as well as other oxidized sterols, exhibited the strongest LXR activating capacity in SH-SY5Y cells, in line with their relatively high uptake (19.65% ± 2.40 for **S1** and 43.74% ± 7.74 for **S6**) (Table S1). In HepG2 cells, the percentage of **S1** internalization was only 1.67% ± 0.34 resulting in a low intracellular concentration (Table S1). **S6** could not even be detected in HepG2 cells, although the concentration in the medium decreased significantly (unpublished data), suggesting a possible conversion into (currently unknown) metabolites. This relatively low internalization may support the observation that only high concentrations of the oxidized sterols can activate LXRs in HepG2 cells.

We did not find any indication of RXR activation by the oxidized sterols in SH-SY5Y and only a limited effect on RXR activation in CHME3 and CCF-STTG1. Because of the limited effect of the compounds on the activation of RXR, our data indicate that the ability of the selected active compounds to modulate *APOE*, *ABCA1*, *ABCG1,* and *SREBF1* expression in CNS cell lines is likely to rely on LXR/RXR heterodimers through the specific activation of the LXR pathway directly in the CNS. However, in HepG2, RXR may also contribute because it was activated by high concentrations of the compounds.

The LXR agonists **S1**, **S2**, **S6**, **N10**, and **N12** were found to induce the expression of the LXR target genes involved in cholesterol trafficking. *APOE* transcription was induced specifically in CCF-STTG1 astroglial cells, and the expression of *ABCA1* and *ABCG1* encoding cholesterol efflux transporters was increased in all CNS cell lines. These data are supportive of the effect of the oxidized sterols on the ApoE-mediated cholesterol turnover in the brain. Cholesterol is synthesized predominantly by astrocytes within the brain as the blood-brain barrier prevents its retrieval from circulation [58,59]. Astrocytes, through the activity of ABCA1 and ABCG1 transports, release cholesterol associated with ApoE-containing- lipoprotein-like particles to provide neurons with cholesterol and other lipids, in order to maintain neuronal physiological functions [60]. However, to what extent the oxidized sterols can reach the brain remains to be determined.

Because **S1** and **S6**, in SH-SY5Y and HepG2 cells increased the concentrations of desmosterol, an endogenous LXR agonist, these compounds may regulate LXR target genes and cholesterol homeostasis through the upregulation of desmosterol production. On the other side, cholesterol concentrations were not increased in HepG2 cells, possibly as a result of increased cholesterol efflux into the medium [12]. However, induction of cholesterol efflux to human serum from HepG2 cells was observed after incubation with **S6**, but not **S1**. Upregulation of desmosterol may result in the activation of LXR and its target genes, the inhibition of SREBP target genes, the selective reprogramming of fatty acid metabolism, and the suppression of inflammatory-response genes, as previously observed in macrophage foam cells [15]. **S1** and **S6** also increased desmosterol concentrations in SH-SY5Y cells, while concentrations of cholesterol as well as lanosterol, and lathosterol were reduced. The notable increase in desmosterol in SH-SY5Y cells may result in LXR activation and consequently, the inhibition of cholesterol synthesis, the increased conversion into 24(S)-hydroxycholesterol and thereby increased cholesterol efflux. All five newly identified candidate LXR-agonists, similar to saringosterol, upregulated the expression of *CYP46A1* about three-fold in SH-SY5Y cells exclusively. In the brain, 24S-hydroxycholesterolacts as a key modulator of both cholesterol homeostasis and inflammatory signaling in the central nervous system (CNS) [10,61]. Upregulation of CYP46A1 has been demonstrated to have beneficial effects for a number of neurodegenerative diseases, including AD, HD and PD [62,63,64]. Although the inhibition of the gene coding for DHCR24 could be underlying the accumulation of desmosterol [65,66], its expression was not affected by **S1** and **S6** in SH-SY5Y nor in HepG2 cells; DHCR7, involved in desmosterol synthesis, was not affected. Therefore, the mechanism underlying the **S1** and **S6**-induced increase in desmosterol remains to be established. On the other hand, **S1** and **S6** may directly inhibit cholesterol synthesis in astrocytes, as cholesterol and its precursors, lanosterol and lathosterol, were reduced while desmosterol concentrations remained unaffected in these cells. Moreover, LXR activation may induce ApoE-mediated cholesterol secretion.

One of the limitations of this study is that the experiments were performed by an in vitro approach using cell lines. Moreover, due to the broad testing panel, the sample size was limited to obtain feasibility.

In summary, we identified five novel LXR-activating oxidized sterols with the potential to regulate cholesterol homeostasis. Although we did not evaluate the anti-inflammatory effects of the compounds, we found a marked impact of **S1** and **S6** on cellular concentrations of desmosterol, which in addition to its key role in the regulation of cholesterol metabolism, has been shown to suppress inflammatory-response genes in macrophage foam cells [15,67]. In addition, these compounds enhanced the neuronal expression of *CYP46A1* with promising effects in different neurodegenerative and cardiovascular diseases. Moreover, the five oxidized sterols activated LXRs without inducing SREBP1c-mediated lipogenesis in hepatocytes, these compounds may have the potential to prevent or delay the progression of Alzheimer’s disease, other neurodegenerative diseases and cardiovascular diseases since their effect is not accompanied by undesirable adverse hepatic effects.

## 4. Materials and Methods

### 4.1. Side Chain Oxidized Cholesterol Derivatives

**Figure 8:**
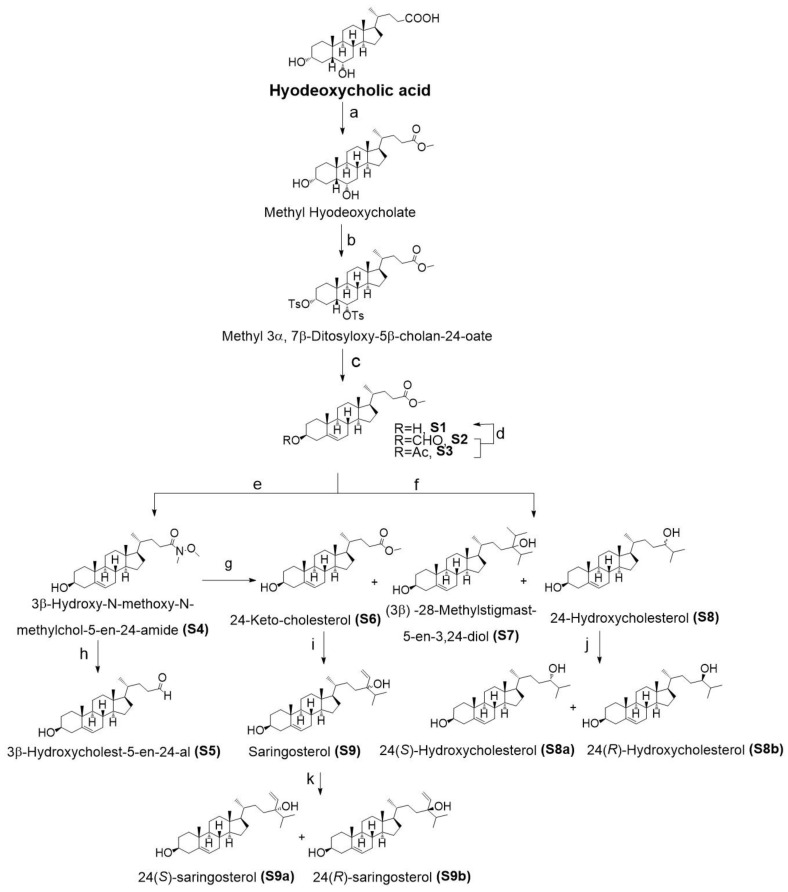
Synthesis of oxidized sterols, reagents and conditions: (**a**) CH_3_OH, H_2_SO_4_, 8 h, 95 °C, 89.0%; (**b**) TsCl, pyridine (Py), 24 h, room temperature (r.t)., 77.9%; (**c**) AcOK, DMF, H_2_O, 7 h, 105 °C, **S1** 50.9%, **S2** 4.2%, **S3** 4.5%; (**d**) 2% KOH-CH_3_OH, 3 h, r.t., 96.7%. (**e**) Isopropylmagnesium chloride solution, N,O-dimethylhydroxylamine hydrochloride, tetrahydrofuran(THF), 12 h, 0 °C-r.t., **S4** 85.5%; (**f**) Isopropylmagnesium bromide solution, triethylamine (Et3N), THF, 18 h, 0 °C-r.t., **S6** 26.8%, **S7** 2.8%, S8 8.2%; (**g**) Isopropylmagnesium chloride solution, THF, 18 h, 0 °C-r.t., **S6** 52.0%; (**h**) Vinylmagnesium chloride solutionchlorovinylmagnesium, THF, 18 h, 0 °C-r.t., S5; (**i**) Isopropylmagnesium chloride solution, THF, 12 h, 0 °C-r.t., S9 90.8%; (**j**) Semi-PHPLC equivalent elution, MeOH-H_2_O, 90:10 (*v/v*), **S8a** and **S8b** in a 1:1 ratio; (**k**) Semi-PHPLC equivalent elution, MeOH- CH_3_CN-H_2_O, 85:1:14 (*v/v/v*), **S9a** and **S9b** in a 1:1 ratio.

The synthesis of oxysterols was started with commercially available hyodeoxycholic acid (UHN Shanghai Research and Development Co., Ltd. Shanghai, China ). The esterification of hyodeoxycholic acid afforded the corresponding methyl hyodeoxycholate, which reacted with tosyl chloride in pyridine to yield the ditosylated ester, methyl 3α, 7β-ditosyloxy-5β-cholan-24-oate [68,69]. Next, in the presence of acetic acid (AcOK) by using N,N-Dimethylformamide (DMF)/H2O as a solvent, alkene methyl 3β-hydroxychol-5-en-24-oate (**S1**) was obtained in 50.9% yield from methyl 3α, 7β-ditosyloxy-5β-cholan-24-oate via S_N_2 displacement and elimination, together with large quantities of acetylated 3β-formyloxychola-5-ene-24-oic acid methyl ester (**S2**) or 3β-acetoxychola-5-ene-24-oic acid methyl ester (**S3**) (Figure 8. An additional in situ hydrolysis step could increase the yield of **S1** to 96.7%. We obtained Weinreb amides **S4** from **S1** using the Grignard reagent isopropylmagnesium chloride, followed by the subsequent addition of isopropylmagnesium chloride to afford the corresponding ketone **S6**. When vinylmagnesium bromide was used instead of isopropylmagnesium chloride to react with **S4**, **S5** was generated. Subsequently, alkene **S1** can react in a two-step sequence through a Weinreb amide formation followed by two consecutive Grignard additions that led to 24-ketocholesterol (**S6**), (3β)-28-methylstigmast-5-en-3,24-diol (**S7**), 24-hydroxycholsterol (**S8**) and saringosterol (**S9**), respectively. Both **S8** and **S9** were obtained as an epimeric mixture in a 1:1 ratio, which was separated by semi-preparative HPLC (semi-PHPLC) to obtain 24(*S*)-epimeric and 24(*R*)-epimeric.

Mass spectrum and ^1^H NMR data of identified compounds:

**Hyodeoxycholic acid:**  ESI-MS: *m/z* 415 [M+Na]^+^; ^1^H NMR (DMSO-*d*_6_, 500 MHz): *δ* 11.95 (1H, s, -COOH), 4.41 (1H, d, *J* = 3.7 Hz, -OH), 4.23 (1H, d, *J* = 3.6 Hz, -OH), 3.82 (1H, d, *J* = 7.6 Hz, H-3), 0.87 (3H, d, *J* = 6.5 Hz, H-21), 0.83 (3H, s, H-19), 0.60 (3H, s, H-18). According to the literature [70], it was identified as Hyodeoxycholic acid.

**Methyl Hyodeoxycholate:**  ESI-MS: *m/z* 429 [M+Na]^+^, 389 [M-H_2_O+H]^+^, 371 [M-2H_2_O+H]^+^; ^1^H NMR (500 MHz, CDCl_3_): *δ* 4.05 (1H, m, H-6), 3.66 (3H, s, 24-OCOCH_3_), 3.61 (1H, m, H-3), 0.91 (3H, d, *J* = 6.5 Hz, H-21), 0.90 (3H, s, H-19), 0.63 (3H, s, H-18). According to the literature [68], it was identified as Methyl Hyodeoxycholate.

**Methyl 3*α*, 7*β*-Ditosyloxy-5*β*-cholan-24-oate:**  ESI-MS: *m/z* 737 [M+Na]^+^; ^1^H NMR (500 MHz, CDCl_3_): *δ* 7.78 (2H, d, *J* = 8.0 Hz, 3*α*- and 6*α*-C_6_H_4_CH_3_), 7.72 (2H, d, *J* = 8.0 Hz, 3*α*- and 6*α*-C_6_H_4_CH_3_), 7.34 (4H, t, *J* = 9.0 Hz, 3*α*- and 6*α*-C_6_H_4_CH_3_), 4.78 (1H, m, H-6), 4.30 (1H, m, H-3), 3.66 (3H, s, 24-OCOCH_3_), 2.46 (6H, s, 3*α*- and 6*α*-C_6_H_4_CH_3_), 2.33 (1H, m), 2.20 (1H, m), 0.88 (3H, d, *J* = 6.4 Hz, H-21), 0.80 (3H, s, H-19), 0.59 (3H, s, H-18). According to the literature [71], it was identified as Methyl 3*α*, 7*β*-Ditosyloxy-5*β*-cholan-24-oate.

**Methyl 3*β*-hydroxychol-5-en-24-oate (S1):**  ESI-MS: *m/z* 411 [M+Na]^+^, 389 [M+H]^+^, 371 [M-H_2_O+H^+^]; ^1^H NMR (500 MHz, CDCl_3_): *δ* 5.35 (1H, br d, H-6), 3.66 (3H, s, 24-OCOCH_3_), 3.52 (1H, m, H-3), 1.00 (3H, s, H-19), 0.92 (3H, d, *J* = 6.5 Hz, H-21), 0.67 (3H, s, H-18). According to the literature [71], it was identified as Methyl 3*β*-hydroxychol-5-en-24-oate.

**3*β*-Formyloxychola-5-en-24-oic acid methyl ester (S2):**  ESI-MS: *m/z* 855 [2M+Na]^+^, 439 [M+Na]^+^; ^1^H NMR (500 MHz, CDCl_3_): *δ* 8.03 (1H, s, 3*β*-OCHO), 5.39 (1H, br d, H-6), 4.73 (1H, m, H-3), 3.66 (3H, s, 24-OCOCH_3_), 1.02 (3H, s, H-19), 0.92 (3H, d, *J* = 6.5 Hz, H-21), 0.68 (3H, s, H-18). According to the literature [71], it was identified as 3*β*-Formyloxychola-5-ene-24-oic acid methyl ester.

**3*β*-Acetoxychola-5-en-24-oic acid methyl ester (S3):**  ESI-MS: *m/z* 883.7 [2M+Na]^+^, 453 [M+Na]^+^; ^1^H NMR (500 MHz, CDCl_3_): *δ* 5.37 (1H, br d, H-6), 4.59 (1H, m, H-3), 3.66 (3H, s, 24-OCOCH_3_), 2.03 (3H, s, 3*β*-OCOCH_3_), 1.02 (3H, s, H-19), 0.92 (3H, d, *J* = 6.4 Hz, H-21), 0.68 (3H, s, H-18). According to the literature [71], it was identified as 3*β*-Acetoxychola-5-ene-24-oic acid methyl ester.

**3*β*-Hydroxy-*N*-methoxy-*N*-methylchol-5-en-24-amide (S4):**  ESI-MS: *m/z* 857 [2M+Na]^+^, 835 [2M+H]^+^, 440 [M+Na]^+^, 418 [M+H]^+^; ^1^H NMR (600 MHz, CDCl_3_): *δ* 5.32 (1H, br d, H-6), 3.67 (3H, s, -OCH_3_), 3.49 (1H, m, H-3), 3.15 (3H, s, -NCH_3_), 0.99 (3H, s, H-19), 0.93 (3H, d, *J* = 6.5 Hz, H-21), 0.665 (3H, s, H-18). According to the literature [72], it was identified as 3*β*-Hydroxy-*N*-methoxy-*N*-methylchol-5-en-24-amide.

**3*β*-Hydroxycholest-5-en-24-al (S5):**  ESI-MS: *m/z* 381.2 [M+Na]^+^; ^1^H NMR (500 MHz, CDCl_3_) δ 9.765 (1H, s, -CHO), 5.347 (1H, br d, H-6), 3.520 (1H, m, H-3), 1.003 (3H, s, H-19), 0.925 (3H, d, *J* = 6.4 Hz, H-21), 0.679 (3H, s, H-18). According to the literature [72], it was identified as 3*β*-Hydroxycholest-5-en-24-al.

**24-ketocholesterol (S6):**  ESI-MS: *m/z* 423 [M+Na]^+^, 401 [M+H]^+^, 383 [M-H_2_O+H]^+^, 365 [M-2H_2_O+H]^+^; ^1^H NMR (500 MHz, CDCl_3_): *δ* 5.35 (1H, br d, *J* = 3.5 Hz, H-6), 3.52 (1H, m, H-3), 2.61 (1H, m, H-25), 1.09 (6H, d, *J* = 6.9 Hz, H-26 and H-27), 1.00 (3H, s, H-19), 0.91 (3H, d, *J* = 6.6 Hz, H-21), 0.67 (3H, s, H-18). According to the literature [73], it was identified as 24-ketocholesterol.

**(3*β*)-28-Methylstigmast-5-en-3,24-diol (S7):**  ESI-MS: *m/z* 427 [M-H_2_O+H]^+^, 409 [M-2H_2_O+H]^+^; ^1^H NMR (500 MHz, CDCl_3_): *δ* 5.35 (1H, br d, H-6), 3.53 (3H, m, H-3), 1.00 (3H, s, H-19), 0.95 (6H, d, *J* = 6.7 Hz, H-26, H-27), 0.94 (3H, d, *J* = 6.9 Hz, H-21), 0.92 (6H, d, *J* = 6.9 Hz, H-29, H-30), 0.677 (3H, s, H-18); ^13^C NMR (125MHz, CDCl_3_): *δ* 140.9 (C-5), 121.8 (C-6), 77.6 (C-24), 72.0 (C-3), 56.9 (C-14), 55.9 (C-17), 50.2 (C-9), 42.5 (C-13), 42.5 (C-4), 24.5 (C-15), 37.4 (C-1), 37.1 (C-20), 34.2 (C-25), 33.8 (C-28), 32.0 (C-7), 31.8 (C-2), 30.9 (C-23), 30.2 (C-22), 39.9 (C-12), 28.5 (C-16), 21.2 (C-11), 19.6 (C-19), 18.8 (C-21), 17.8 (C-29), 17.7 (C-30), 17.4 (C-26), 17.3 (C-27), 12.0 (C-18), 32.1 (C-8), 36.7 (C-10). It was identified as (3*β*) -28-Methylstigmast-5-en-3,24-diol.

**24-Hydroxycholesterol (S8):**  ESI-MS: *m/z* 385 [M-H_2_O+H]^+^, 367 [M-2H_2_O+H]^+^; ^1^H NMR (500 MHz, CDCl_3_): *δ* 5.35 (1H, m, H-6), 3.53 (1H, m, H-3), 3.31 (1H, br s, H-24), 1.01 (3H, s, H-19), 0.93 (6H, m, H-26, H-27), 0.91–0.89 (3H, m, H-21), 0.68 (3H, s, H-18). According to the literature [74], it was identified as 24-Hydroxycholesterol.

**24S-Hydroxycholesterol (S8a):**  APCI-MS: *m/z* 385.4 [M-H_2_O+H]^+^, 367.4 [M-2H_2_O+H]^+^; ^1^H NMR (CDCl_3_, 500 MHz) δ 5.351 (1H, d, *J* = 5.0 Hz, H-6), 3.532 (1H, m, H-3), 3.308 (1H, br s, H-24), 1.006 (3H, s, H-19), 0.932 (6H, t, *J* = 6.2 Hz, H-26, H-27), 0.896 (3H, d, *J =* 6.8 Hz, H-21), 0.679 (3H, s, H-18). According to the literature [74,75], it was identified as 24S-Hydroxycholesterol

**24R-Hydroxycholesterol (S8b):**  ESI-MS: *m/z* 385 [M-H_2_O+H]^+^, 367 [M-2H_2_O+H]^+^; ^1^H NMR (500 MHz, CDCl_3_) δ 5.351 (1H, m, H-6), 3.529 (1H, m, H-3), 3.316 (1H, s, H-24), 1.006 (3H, s, H-19), 0.926 (6H, m, H-26, H-27), 0.907 (3H, d, *J =* 2.8 Hz, H-21), 0.684 (3H, s, H-18). According to the literature [74], it was identified as 24R-Hydroxycholesterol

**Saringosterol (S9):**  APCI-MS: *m/z* 411 [M-H_2_O+H]^+^, 393 [M-2H_2_O+H]^+^; ^1^H NMR (500 MHz, CDCl_3_): δ 5.86–5.75 (1H, m, H-28), 5.35 (1H, br d, H-6), 5.22–5.16 (1H, m, H-29), 5.16–5.11 (1H, m, H-29), 3.52 (1H, m, H-3), 1.00 (3H, s, H-19), 0.92 (3H, m, H-21), 0.89 (3H, m, H-27), 0.87 (3H, d, *J* = 6.9 Hz, H-26), 0.67 (3H, s, H-18). According to the literature [76], it was identified as saringosterol.

**24*S*-saringosterol (S9a):**  APCI-MS: *m/z* 411 [M-H_2_O+H]^+^, 393 [M-2H_2_O+H]^+^; ^1^H NMR (500 MHz, CDCl_3_): *δ* 5.796 (1H, dd, *J* = 17.4, 10.9 Hz, H-28), 5.350 (1H, br d, H-6), 5.184 (1H, dd, *J* = 17.4, 1.0 Hz, H-29), 5.131 (1H, d, *J* = 10.9, 1.0 Hz, H-29), 3.523 (1H, m, H-3), 1.005 (3H, s, H-19), 0.919 (3H, d, *J* = 6.6 Hz, H-21), 0.899 (3H, d, *J* = 6.8 Hz, H-27), 0.872 (3H, d, *J* = 6.9 Hz, H-26), 0.672 (3H, s, H-18). According to the literature [43,77], it was identified as 24*S*-saringosterol.

**24*R*-saringosterol (S9b):**  APCI-MS: *m/z* 411 [M-H_2_O+H]^+^, 393 [M-2H_2_O+H]^+^; ^1^H NMR (500 MHz, CDCl_3_): *δ* 5.810 (1H, dd, *J* = 17.4, 10.9 Hz, H-28), 5.349 (1H, br d, H-6), 5.190 (1H, dd, *J* = 17.4, 1.0 Hz, H-29), 5.137 (1H, d, *J* = 10.9,1.0 Hz, H-29), 3.524 (1H, m, H-3), 1.005 (3H, s, H-19), 0.924 (3H, d, *J* = 6.4 Hz, H-21), 0.891 (3H, d, *J* = 6.8 Hz, H-27), 0.871 (3H, d, *J* = 7.0 Hz, H-26), 0.671 (3H, s, H-18). According to the literature [43,77], it was identified as 24*R*-saringosterol.

### 4.2. Phytosterol Separation from Seaweed

Briefly, air-dried *S. fusiforme* (8 kg) was powdered and extracted with 80% ethanol to obtain a crude extract (550 g) of total lipid. After saponification and extraction, we obtained the phytosterol fraction TS1 (10.06 g), which was fractionated by vacuum liquid chromatography over silica gel by gradient elution using petroleum ether/ethyl acetate to yield eight fractions. The fifth fraction TS1-5 (petroleum ether /ethyl acetate (92:8), 1066.1 mg) was subsequently separated over flash silica gel column chromatography with dichloromethane (DCM)/ petroleum ether (95:5 to 100:0) and DCM / MeOH (50:50) to achieve five subfractions. Subfraction TS15-4 (565.5 mg) was further purified by semipreparative HPLC (MeOH/H_2_O, 90:10) to obtain compound **N10** (18.1 mg), **N11** (14.9 mg), **N12** (27.5 mg), **N13** (3.5 mg).

**(3β,22E)-3-Hydroxycholesta-5,22-dien-24-one (N10):**  ESI-MS: *m/z* 399 [M+H]^+^, 381[M-H_2_O+H]^+^; ^1^H NMR (500 MHz, CDCl_3_): *δ* 6.71 (1H, dd, *J* = 15.5, 9.0 Hz, H-22), 6.07 (1H, d, *J* = 16 Hz, H-23), 5.35 (1H, *brd*, H-6), 3.53 (1H, m, H-3), 2.83 (1H, m, H-25), 1.10 (6H, d, *J* = 6.5 Hz, H-26,27), 1.10 (3H, d, *J* = 6.5 Hz, H-21), 1.01 (3H, s, H-19), 0.72 (3H, s, H-18). According to the literature [73], it was identified as (3β,22E)-3-Hydroxycholesta-5,22-dien-24-one.

**(3β,23E)-3-Hydroxystigmasta-5,23-dien-28-one (N11):**  ESI-MS: *m/z* 427[M+H]^+^, 409[M-H2O+H]^+^; ^1^H NMR (500 MHz, CDCl_3_): *δ* 6.45 (1H, dd, *J* = 7.85, 6.75 Hz, H-23), 5.35 (1H, *brd*, H-6), 3.53 (1H, m, H-3), 2.87 (1H, m, H-25), 2.34 (1H, m, H-22a), 2.27 (3H, s, H-29), 2.03 (1H, m, H-22b), 1.14 (2H, d, *J* = 7.0 Hz, H-26, 27), 1.01 (3H, s, H-19), 0.96 (3H, d, *J* = 6.6 Hz, H-21), 0.72 (3H, s, H-18). According to the literature [78], it was identified as (3β,23E)-3-Hydroxystigmasta-5,23-dien-28-one.

**Fucosterol-24,28 epoxide (N12):**  ESI-MS: *m/z* 429[M+H]^+^, 411[M-H2O+H]^+^; 5.35(1H, *brd,* H-6), 3.53 (1H, m, H-3), 2.878 (1H, q, *J* = 5.6 Hz, H-28), 2.02 (1H, m, H-20), 1.244 (3H, d, *J =* 5.8Hz, H-29), 1.00 (3H, *s,* H-19), 0.914 (3H, d, *J* = 6.4Hz, H-21), 0.892 (3H, d, *J =* 7.2Hz, H-27), 0.863 (3H, d, *J =* 7.2Hz, H-26), 0.68 (3H, *s,* H-18); ^13^C NMR (125MHz, CDCl_3_): *δ* 140.91 (C-5), 121.81 (C-6), 71.94 (C-3), 66.47 (C-24), 57.09 (C-14), 56.87 (C-28), 56.0 (C-17), 50.24 (C-9), 42.5 (C-13), 42.45 (C-4), 39.91 (C-12), 37.4 (C-1), 36.65 (C-20), 36.52 (C-10), 32.22 (C-25), 32.04 (C-8), 32.04 (C-7), 31.81 (C-2), 31.42 (C-22), 25.61 (C-23), 28.39 (C-16), 24.44 (C-15), 21.22 (C-11), 19.55 (C-19), 18.77 (C-21), 18.60 (C-27), 18.18 (C-26), 14.45 (C-29), 12.0 (C-18). According to the literature [79], it was identified as Fucosterol-24,28 epoxide.

**(23Z)-Stigmasta-5,23-diene-3β, 28ξ-diol (N13):**  ESI-MS: *m/z* 429[M+H]^+^, 411[M-H2O+H]^+^; ^1^H NMR (500 MHz, CDCl_3_): *δ*5.49 (1H, *dd*, *J* = 8.29, 6.42 Hz, H-23), 5.35 (1H, *brd*, H-6), 5.24 (1H, *dd*, *J* = 8.25, 6.42 Hz, H-23), 4.76(1H, *q*, *J* = 6.42Hz, H-28), 4.32(1H, *q*, *J* = 6.42Hz, H-28), 3.52 (1H, m, H-3), 2.76 (1H, m, H-25), 2.49 (1H, m, H-25), 1.27(3H, d, *J* = 6.42 Hz, H-29), 1.07 (3H,d, *J* = 6.42 Hz, H-27), 1.04 (3H,d, *J* = 6.42 Hz, H-26), 1.01 (3H, s, H-19), 0.88(3H, d, *J* = 6.42 Hz, H-21), 0.69 (3H, s, H-18). According to the literature [80], it was identified as (23Z)-Stigmasta-5,23-diene-3β, 28ξ-diol.

### 4.3. Cell Culture and Transfection

HEK 293 (human kidney), CCF-STTG1 (human astrocytoma), and SH-SY5Y (human neuroblastoma) cells were obtained from the European Collection of Authenticated Cell Cultures. CHME3 (Human microglial) cells were a kind gift from Prof. Dr. M. Tardieu. All cells were routinely maintained at 37 °C with 5% CO_2_ in a humidified incubator and cultured in Dulbecco’s Modified Eagle Medium/Nutrient Mixture F-12 with Glutamax (DMEM/F-12) supplemented with 10% fetal calf serum (FCS, Gibco Origene, Rockville, USA) and 1% Penicillin/Streptomycin (P/S, Gibco Origene, Brazil). The routine passaging of cells was conducted every week and cells were seeded at 2 × 10^6^ live cells per 250 mL flask to maintain a confluence between 20% and 80%.

### 4.4. Reporter Assays

The functional activity of the candidate LXR ligands was determined in a cell-based transactivation assay. A total of 5.5 × 10^5^ cells in 4 mL were plated onto a T-25 dish the day prior to transfection. Cells were transiently transfected with 1000 ng of LXR (LXRα, LXRβ) expression plasmid, 4000 ng of LXRE-luciferase reporter plasmid and 1000 ng of plasmid encoding RXR in 500 µL DMEM/F-12 using FuGENE^®^ 6 reagent (Promega, Madison, USA) per T-25 dish. The empty pcDNA3.1/V5-HisA vector (Invitrogen, Carlsbad, USA) was used to equalize the total amount of DNA transfected in the blank control condition. Renilla (1000 ng/μL, pRL TK-Renilla) was co-transfected in all experiments to normalize for variation in transfection efficiency [81]. After 24 h, the cells were trypsinized and seeded in 96-well plates and incubated for 24 h in 10% FCS containing DMEM/F-12. Cells were then incubated for 24 h in phenol-red free DMEM/F-12 containing 10% stripped FCS and increasing concentrations of the compound tested or vehicle (DMSO or EtOH). Cells were incubated for 24 h in the presence of indicated concentrations of the compound. Cells were lysed in 100 µL of lysis buffer after 24 h incubation. Firefly luciferase and Renilla luciferase activities were measured with 25 µL of cell lysate using the Dual-Luciferase Reporter assay system (Promega) in a luminometer (Perkin Elmer Victor X4 Multiple plate Reader). ‘Relative activity’ was defined as the ratio of firefly luciferase activity to Renilla luciferase activity and was calculated by dividing the luminescence intensity obtained in the assay for firefly luciferase by that obtained for Renilla luciferase. ‘Fold change’ is defined as the ratio of the relative activity seen with each test compound to the basal relative activity measured in the vehicle control.

For each compound in each cell line, one experiment consisted of performing the stimulation and expression assay in triplicate on three wells of cultured cells independently stimulated in parallel with two or three individually prepared aliquots of transfection reaction.

### 4.5. Quantitative PCR

Total RNA was extracted using Trizol reagent (Invitrogen) according to the manufacturer’s instructions. For all RNA samples, quantity and purity were determined by absorbance at 260 and 280 nm using the Nanodrop ND-1000 spectrophotometer (Nanodrop Technologies, Wilmington, NC, USA).

Contamination of genomic DNA was removed from total RNA samples by dsDNase digestion prior to first-strand synthesis. cDNA synthesis was performed with the Thermo Scientific Maxima H Minus First Strand cDNA Synthesis Kit (#K1681, Thermo Fisher Scientific, Waltham, MA, USA), according to the manufacturer’s instructions.

QPCR was performed using a CFX384 Opus Real-Time PCR Systems (Bio-Rad Laboratories Inc., Veenendaal, the Netherlands. The reactions were carried out in duplicate using Green-based PCR Select master-mix (catlog # 4472903, Thermo Fisher Scientific), following the manufacturer’s instructions. Each reaction was performed in a final volume of 10 μL, primers were used at the concentration of 200 nM. The Thermocycler program consisted of an initial hot start cycle at 50 °C for 2 min followed by 10 min 95 °C. The next steps were 40 cycles at 95 °C for 15 s and 60 °C for 30 s. To confirm product specificity, a melting curve analysis was performed after each amplification.

The exon-exon spanning primers, including four stable reference genes, (Table 2) were designed using the primer design tool in NCBI (http://www.ncbi.nlm.nih.gov/tools/primer-blast/, accessed on 24 November 2022) The efficiency of the primers was calculated by amplifying six serial 1/2 dilutions of each gene amplicon. A standard curve of quantification cycle (Cq) values versus log concentration were plotted to obtain efficiency. Relative gene expression was analyzed using the 2^−ΔΔCt^ method.

### 4.6. Quantitative Analysis of Cholesterol and Cholesterol’s Precursors

Cells (HepG2, CCF-STTG1, and SH-SY5Y) were plated in 12-well dishes and incubated with the specified concentration of **S1** and **S6** for 24 h. Ethanol-treated cells were used as a control. Total cell sterols were extracted from the cells. Cholesterol, lanosterol, lathosterol, and desmosterol were determined from these cell samples using GC/MS as described previously [82,83]. Briefly, fifty micrograms of 5α-cholestane (Serva) (50 μL from a stock solution in cyclohexane; 1 mg/mL), 1 μg epicoprostanol (Sigma-Aldrich, St Louis, MO, USA) (10 μL from a stock solution in cyclohexane; 100 μg/mL), were added as internal standards to an aliquot of cells. Sterols were extracted by cyclohexane after saponification and neutralization. The solvents were evaporated and the residual sterols and oxysterols were derivatized to trimethylsilyl (TMSi)-ethers by adding 300 µL TMSi-reagent (pyridine-hexamethy43ldisilazane-Chlorotrimethylsilane; 9:3:1, *v/v/v*; all reagents were supplied by Merck, Darmstadt, Germany) and incubated for 2 h at 90 °C. The solvents were evaporated under nitrogen at 65 °C. The pellet was dissolved in 80 μL n-decane and was transferred into a micro-vial for gas-liquid chromatographic-mass spectrometric (GC-MS) analysis of cholesterol precursors and sterols.

### 4.7. Cholesterol Efflux Studies

The effect of selected oxidized sterols on cellular cholesterol efflux was evaluated in the HepG2 cell line by a standardized radioisotopic technique. After plating, HepG2 cells were labeled for 24 h with [1,2-^3^H] cholesterol (PerkinElmer, Milano, Italy) at 2μCi/mL in the presence of 1% fetal calf serum (FCS) containing MEM; 2 μg/mL of an inhibitor of acyl-coenzyme A: cholesterol acyltransferase (ACAT, Sandoz 58035; Sigma-Aldrich) were added to prevent the accumulation of cholesteryl esters. Cell monolayers were then equilibrated for 20 h in 0.2% Bovine serum albumin-containing medium (BSA from Sigma-Aldrich, St. Louis, MO, USA), in presence of ACAT inhibitor, in basal condition, or supplemented with T0901317 (Cayman Chemical Company, Ann Arbor, MI, USA) at 1 µM, or increasing concentrations of compounds. Subsequently, efflux of cholesterol was induced by incubation for 4 h with 2% (*v/v*) serum of a pool of normolipidemic subjects, 10 µg/mL of lipid-free human apolipoprotein A-I (Sigma-Aldrich, St Louis, MO, USA) or 12.5 µg/mL of human High-Density Lipoprotein (HDL, from Sigma-Aldrich, St Louis, MO, USA) in the medium. To prevent the remodeling of the lipoproteins, sera were slowly thawed in ice immediately prior to this procedure. Cholesterol efflux was expressed as a percentage and obtained by measuring the release of radiolabeled cholesterol into the medium over the total radioactivity incorporated by cells. To correct for the inter-assay variability, the HDL-cholesterol efflux capacity (CEC) percentage of basal and T0901317 conditions were used to normalize the different experiments. Intra-assay Coefficient of Variation (CV) for HDL-CEC assays was <10%.

### 4.8. Statistical Analysis

All statistical analyses were performed using GraphPad Prism 8. The experiments were performed as three independent experiments. Data are presented as mean ± standard deviation (SD) for parameters with normal distribution. The fold change values relative to the DMEM/F-12 EtOH or DMSO control were analyzed using ordinary One way ANOVA with a posthoc Dunnet correction for multiple comparisons. The levels of significance are denoted as follows (alpha = 0.05): * *p* ≤ 0.05, ** *p* ≤ 0.01, *** *p* ≤ 0.001.

## 5. Conclusions

In the quest of identifying LXR-activating sterols with oxygenated sidechains, we identified five novel candidate LXR ligands. These ligands are potential novel regulators of cellular sterol homeostasis. By activating LXRs, they can activate cholesterol efflux and elimination pathways, potentially via enhancing the ApoE-mediated lipid trafficking between astrocytes and neurons. The identified ligands also have beneficial effects in enhancing cholesterol turnover in the brain by upregulating *CYP46A1* expression. Moreover, **S1** and **S6** regulate the endogenous LXR ligand desmosterol concentration, which plays a key role in the regulation of cholesterol metabolism and can suppress inflammatory-response genes in macrophages. In contrast to most synthetic LXR-agonists, the five novel LXR-activating oxidized sterols are anticipated not to induce the unwanted side effects of lipid accumulation in the liver and in the circulation due to their auto-regulated secretion in the liver. The novel LXR-activating oxysterols are, therefore, promising for the prevention and for slowing down the progression of neurodegenerative diseases, including AD, and may be useful in the treatment of cardiovascular diseases.

## Figures and Tables

**Table 1:** LXRα and β transcriptional activities of side-chain oxysterols in different cell lines.

Cell line	HEK293		CCF-STTG1		SH-SY5Y		CHME3		HepG2			
**LXR**	α	β	α	β	α	β	α	β	α	β		
**Compouds**	5.0μM											
**S1**	2.69	3.29	1.73	1.37	7.85	6.30	1.89	2.26	2.56	2.33		
**S2**	1.90	1.80	0.76	1.32	5.44	5.09	1.75	1.24	2.40	2.55		
**S3**	2.72	1.74	1.39	1.51	1.97	1.74	1.46	1.28	1.05	1.39	**Fold change**	
**S4**	1.62	1.25	1.34	1.45	0.19	0.33	1.51	1.98	0.82	0.80		3.5 < X
**S5**	1.33	0.99	1.07	1.16	0.24	0.45	1.13	1.05	0.67	0.71		3.0 < X ≤ 3.5
**S6**	2.34	2.82	2.28	2.87	9.90	7.26	1.87	2.67	3.22	2.73		2.5 < X ≤ 3.0
**S7**	1.67	1.53	1.30	1.12	1.08	1.33	1.24	1.39	0.83	1.45		2.0 < X ≤ 2.5
**S8a**	2.51	2.61	1.72	2.25	7.79	6.90	1.73	2.40	2.64	3.70		1.5 < X ≤ 2.0
**S8b**	2.07	2.12	1.93	2.45	3.56	2.72	1.51	1.39	1.54	2.01		1.0 < X ≤ 1.5
**S9a**	1.55	1.96	1.26	2.12	4.40	3.77	1.70	2.01	2.34	2.75		≤1.0
**S9b**	1.17	1.22	0.62	0.49	0.96	1.87	1.14	1.07	1.37	1.14		
**N10**	2.86	3.67	0.86	0.91	12.56	8.82	2.20	2.75	2.88	4.15		
**N11**	1.55	1.45	1.02	1.05	2.56	2.36	1.40	1.77	1.87	2.30		
**N12**	2.17	2.57	0.91	1.30	7.89	7.71	2.47	3.05	1.38	4.23		
**N13**	1.21	0.86	1.00	1.32	0.09	0.20	1.33	1.66	0.37	0.59		

**Table 2:** Sequence of the primers used for quantitative PCR.

Gene (Human)	Forward	Reverse	Order/Design Date
*APOE*	ACCCAGGAACTGAGGGC	CTCCTTGGACAGCCGTG	13 November 2017
*ABCA1*	TCTCTGTTCGGCTGAGCTAC	TGCAGAGGGCATGGCTTTAT	26 June 2017
*ABCG1*	GGTCGCTCCATCATTTGCAC	GCAGACTTTTCCCCGGTACA	26 June 2017
*SREBF1*	ACAGCCATGAAGACAGACGG	CAAGATGGTTCCGCCACTCA	15 September 2020
*ACACA*	GGGTCAAGTCCTTCCTGCTC	GGACTGTCGAGTCACCTTAAGTA	30 August 2022
*FASN*	CACAGACGAGAGCACCTTTGA	CAGGTCTATGAGGCCTATCTGG	22 October 2019
*SCD1*	GCTGTCAAAGAGAAGGGGAGT	AGCCAGGTTTGTAGTACCTCCT	10 May 2021
*NR1H3* (LXRA)	GTTATAACCGGGAAGACTTTGC	AAACTCGGCATCATTGAGTTG	29 August 2018
*NR1H2* (LXRB)	AAGCAAGTGCCTGGTTTCCT	GCAGCATGATCTCGATAGTGGA	26 June 2017
*DHCR7*	TGGGCCAAGACTCCACCTAT	ACGTGTACAGAAGCACCTGG	12 July 2021
*DHCR24*	GTCTCACTACGTGTCGGGAA	CTCCACACGGACAATCTGTTTC	10 May 2021
*CYP27A1*	GGGCAAGTACCCAGTACGGA	TGGTGTCCTTCCGTGGTGAA	8 June 2021
*CYP46A1*	TGTGTTTGGTGAGAGACTCTTCG	GCCAGGTCTATGACTCTCCG	14 October 2020
*HPRT1*	TGACACTGGCAAAACAATGCA	GGTCCTTTTCACCAGCAAGCT	10 February 2017
*B2M*	CTCCGTGGCCTTAGCTGTG	TTTGGAGTACGCTGGATAGCCT	10 February 2017
*SDHA*	TGGGAACAAGAGGGCATCTG	CCACCACTGCATCAAATTCATG	12 May 2011
*ACTB*	CTCCCTGGAGAAGAGCTACG	GAAGGAAGGCTGGAAGAGTG	12 May 2011

## Data Availability

The data that support the findings of this study are available from the corresponding author upon reasonable request.

## Supplementary Materials

The following supporting information can be downloaded at: https://www.mdpi.com/article/10.3390/ijms24021290/s1.
