# Supplementary material for: Identification of Side Chain Oxidized Sterols as Novel Liver X Receptor Agonists with Therapeutic Potential in the Treatment of Cardiovascular and Neurodegenerative Diseases
Source: Int J Mol Sci. 2023 Jan 9;24(2):1290. doi: 10.3390/ijms24021290 (PMC9863018; doi:10.3390/ijms24021290)
Supplement: Supplementary file 1 [file ijms-24-01290-s001.zip › ijms-2089137-supplementary.pdf]

Supplementary Materials

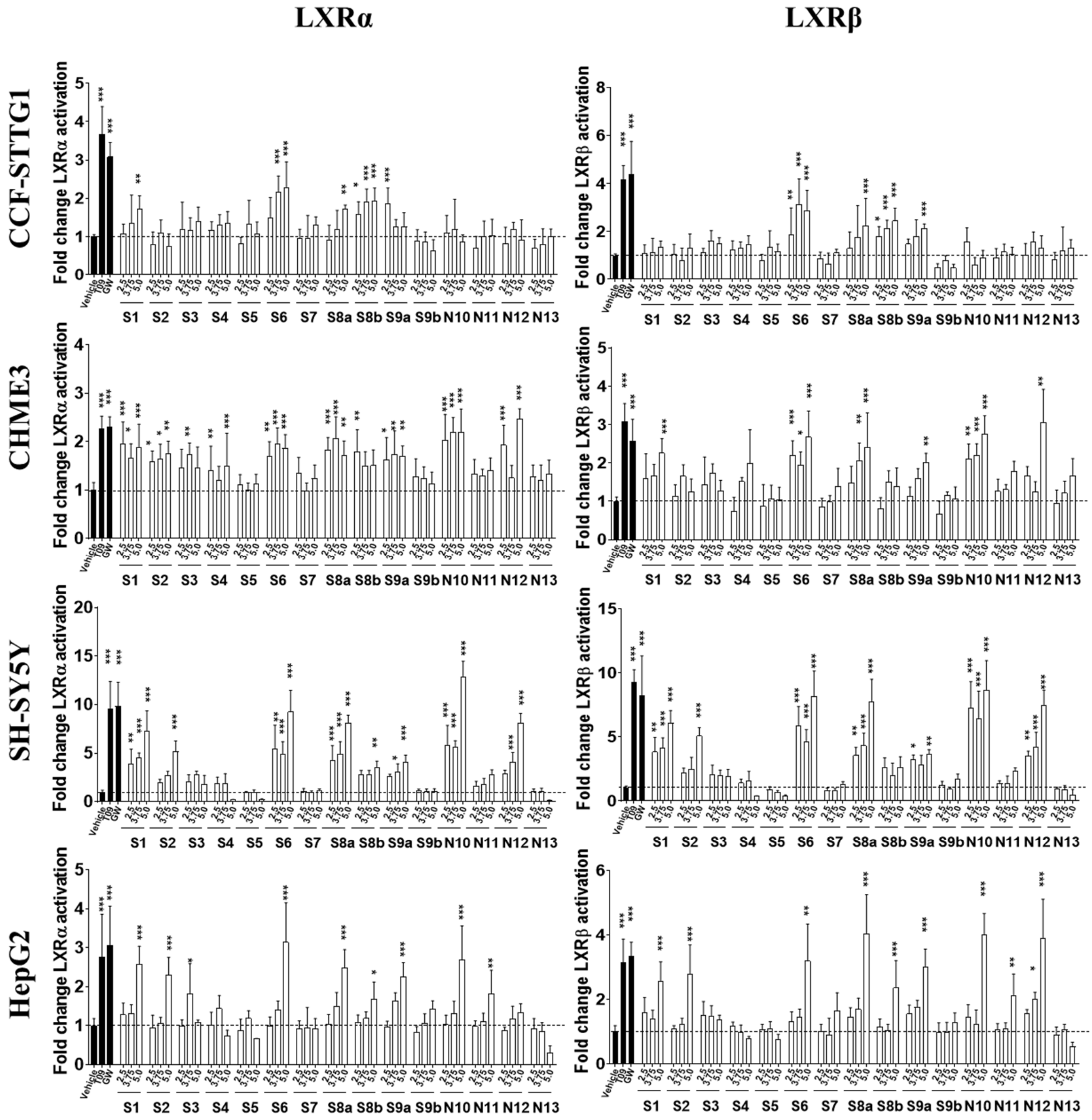

Figure S1. Effects of side chain oxidized sterols on transcriptional activity of LXRα and β in different cell lines. Using the luciferase reporter assay the capacity of oxidized sterols on LXR-mediated transcription was determined. Transfected cells were incubated with different concentrations of oxysterols (2.5, 3.75 and 5.0 μM) for 24h. T0901317 (T09, 1μM) and GW3965 (GW, 5μM) were used as positive controls. Data represent the mean ± SD of three separate experiments, each performed in triplicate (*n*=9). Significance is compared to the control (DMEM/F-12 medium with EtOH or DMSO) value: * $p \leq 0.05$, ** $p \leq 0.01$, *** $p \leq 0.001$.

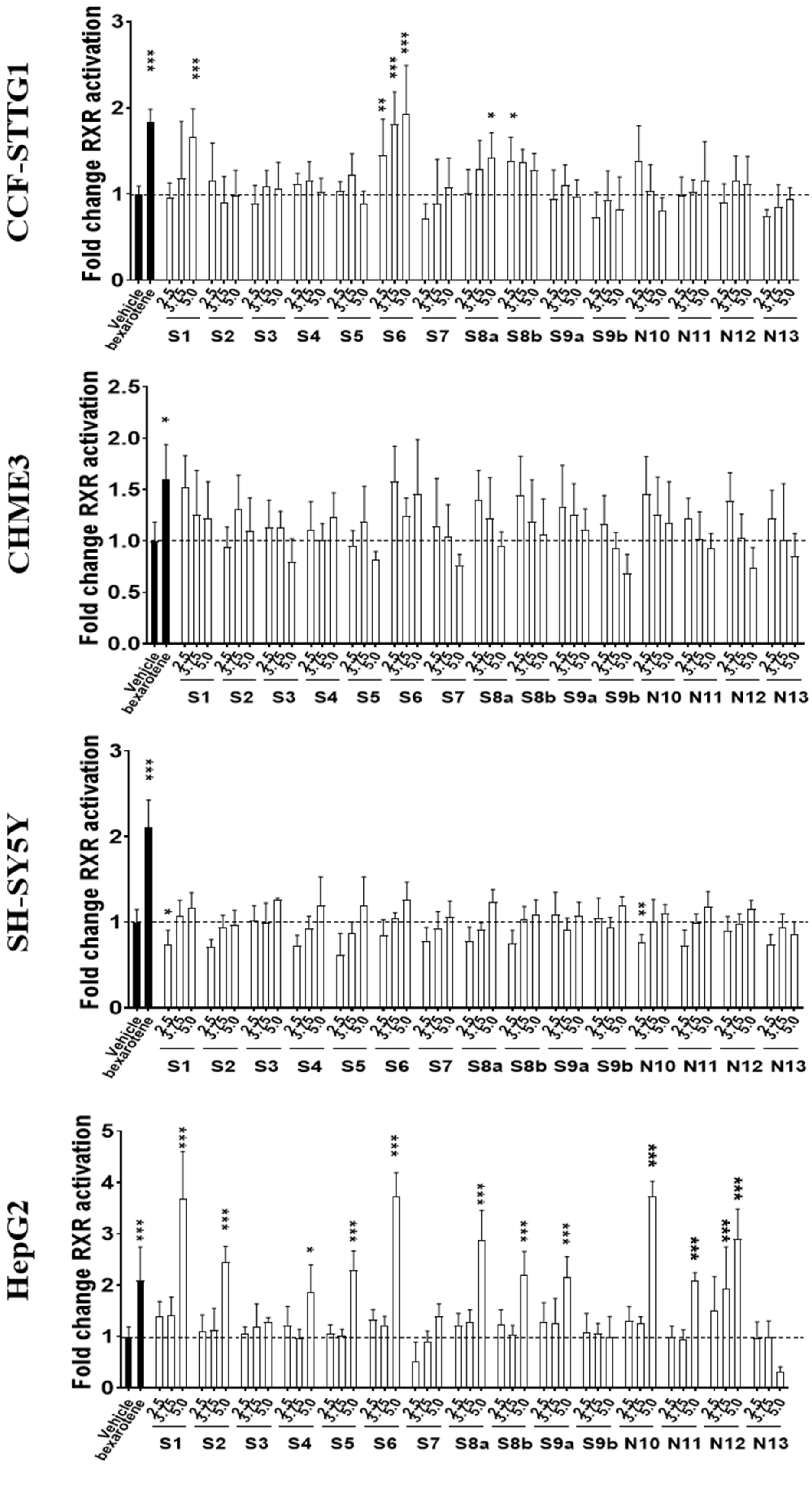

Figure S2. Effects of side chain oxidized sterols on RXR activation. Using the luciferase reporter assay the capacity of oxidized sterols on RXR-mediated transcription were determined.

Transfected cells were incubated with different concentrations of oxysterols (2.5, 3.75, and 5.0 µM) for an 24h. Bexarotene (0.1µM) served as positive controls. Data represent the mean ± SD of three separate experiments, each performed in triplicate (*n*=9). Significance is compared to the control (DMEM/F-12 medium with EtOH or DMSO) value: * $p \le 0.05$, ** $p \le 0.01$, *** $p \le 0.001$.

Table S1 The percentage of **S1** or **S2** was internalized by cells

Compounds	**Remain in the medium (%)**			**Internalization into cells (%)**		
	SH-SY5Y	CCF-STTG1	HepG2	SH-SY5Y	CCF-STTG1	HepG2
**S1**	80.45±2.43	98.69±3.10	98.21±2.84	19.65±2.08	0.29±0.20	1.79±0.40
**S6**	56.26±4.93	80.78±3.76	/	43.74±7.47	19.22±3.51	/

Note: 2.5 µM **S1** or **S6** was loaded to cells (HepG2, CCF-STTG1 and SH-SH5Y) and incubated 24h. Data represent the mean ± SD of three separate experiments, each performed in triplicate (*n*=9)
